# Emergency infection prevention and control training in fragile, conflict-affected or vulnerable settings: a scoping review

**DOI:** 10.1186/s12913-024-11408-y

**Published:** 2024-08-16

**Authors:** Julii Brainard, Isabel Catalina Swindells, Joanna Wild, Charlotte Christiane Hammer, Emilio Hornsey, Hibak Osman Mahamed, Victoria Willet

**Affiliations:** 1 Norwich Medical School University of East, Anglia Norwich, UK; 2 UCL Medical School, University College London, London, UK; 3 OL4All, Oxford, UK; 4 Department of Veterinary Medicine, University of Cambridge, Cambridge, UK; 5 London School of Hygiene & Tropical Medicine, UK Public Health Rapid Support Team, UK Health Security Agency, and , London, UK; 6 Country Readiness Strengthening, World Health Organization, Geneva, Switzerland

**Keywords:** Infections, Emergency, Training, Infection-control, Education

## Abstract

**Background:**

It is uncertain what could be the best training methods for infection prevention and control when an infectious disease threat is active or imminent in especially vulnerable or resource-scarce settings.

**Methods:**

A scoping review was undertaken to find and summarise relevant information about training modalities, replicability and effectiveness of IPC training programmes for clinical staff as reported in multiple study designs. Eligible settings were conflict-affected or in countries classified as low-income or lower-middle income (World Bank 2022 classifications). Search terms for LILACS and Scopus were developed with input of an expert working group. Initially found articles were dual-screened independently, data were extracted especially about infection threat, training outcomes, needs assessment and teaching modalities. Backwards and forwards citation searches were done to find additional studies. Narrative summary describes outcomes and aspects of the training programmes. A customised quality assessment tool was developed to describe whether each study could be informative for developing specific future training programmes in relevant vulnerable settings, based on six questions about replicability and eight questions about other biases.

**Findings:**

Included studies numbered 29, almost all (*n* = 27) were pre-post design, two were trials. Information within the included studies to enable replicability was low (average score 3.7/6). Nearly all studies reported significant improvement in outcomes suggesting that the predominant study design (pre-post) is inadequate to assess improvement with low bias, that any and all such training is beneficial, or that publication bias prevented reporting of less successful interventions and thus a informative overview.

**Conclusion:**

It seems likely that many possible training formats and methods can lead to improved worker knowledge, skills and / or practice in infection prevention and control. Definitive evidence in favour of any specific training format or method is hard to demonstrate due to incomplete descriptions, lack of documentation about unsuccessful training, and few least-biased study designs (experimental trials). Our results suggest that there is a significant opportunity to design experiments that could give insights in favour of or against specific training methods. “Sleeping” protocols for randomised controlled trials could be developed and then applied quickly when relevant future events arise, with evaluation for outcomes such as knowledge, practices, skills, confidence, and awareness.

**Supplementary Information:**

The online version contains supplementary material available at 10.1186/s12913-024-11408-y.

## Background

A survey of health and care workers in low or lower middle countries in 2017–18 suggested that infection prevention and control (IPC) training while in post was unusual in many countries (reported in 54% of respondent countries [1]). Moreover, such training may only happen when there is already a defined infectious threat present or likely to arrive imminently. A highly responsive strategy in developing and delivering IPC training means opportunity to customise training formats and methods for local workforce contexts and curricula with regard to very specific pathogens and transmission pathways. However, the context of needing to deliver training urgently with little advance notice of specific pathogen or local context means that such training may be designed and delivered hurriedly, and with minimal setting-specific needs assessment and little evaluation for effectiveness.

As part of past pandemic recovery and future pandemic preparedness, it is useful to collate evidence about which IPC training methods have been applied in specific settings or contexts. Evidence would be especially useful that could be used to inform ongoing development of best training delivery guidelines in settings that may be described as fragile, conflict-affected or otherwise vulnerable (FCV). Best quality evidence may be defined with regard to completeness of reporting (if the training methods are replicable) as well as evidence of effectiveness (desired outcomes). We searched on Google Scholar and Prospero in August 2023 for completed or registered systematic or scoping reviews addressing the topic of emergency IPC training in vulnerable settings. The most similar and comprehensive existing systematic review (Nayahangan et al. 2021; [2]) described medical and/or nursing training (delivered for any clinical training purpose, not just IPC) delivered during viral epidemics (only). The search date for the Nayahangan et al. review was April 2020, more than 3 years before our own study commenced. Systematic literature reviews may be considered ‘out of date’ by two years after their most recent search date [3]. Nayahangan et al. included clinical settings in any country and was not confined to training delivered in emergency or urgent contexts (readiness or response phases [4, 5]). Nayahangan *et. al*. performed quality assessment using the Educational Interventions Checklist [6] which focuses on replicability and mapping of reported teaching methods in the primary research, but only indirectly addresses effectiveness. Nayahangan *et. al*. concluded that previous studies had used a variety of training methods and settings but few training methods had been related to specific patient or other epidemic outcomes. Another somewhat similar previous systematic review was Barrera-Cancedda et al. [7] which described and assessed IPC training strategies in sub-Saharan Africa for nurses. Most of the strategies they found and described were during “business as usual” conditions, rather than readiness or response phases of an outbreak or epidemic presenting imminent threat. Their quality assessment tools were for assessing bias in effectiveness rather than replicability. Their focus was narrowly on nurses in a specific geographic region. Their conclusions arose from considering evidence that went far beyond staff training methods. Barrera-Cancedda et al. concluded that creating good future guidelines for evidence-based practice required that additional primary research to be undertaken from an implementation science-specific perspective.

A challenge in emergency IPC training manifest during the Covid-19 pandemic is inherent to other emerging diseases: early in an outbreak situation there is often uncertainty about the best IPC practices. The actual best practices may vary according to predominant disease transmission pathway(s) that are not yet well-understood. There is merit in considering evidence according to what disease(s) are being prepared for.

This study aimed to provide an updated evidence summary about IPC training formats and apparent effectiveness in a scoping review design. We collected and summarised evidence about IPC training formats and methods as delivered in FCV settings when there was an active infectious disease present (response phase) or the infection arrival was fairly imminent (expected within 6 months, readiness phase) [4, 5]. We undertook a scoping review of IPC training programmes reported in peer reviewed scientific literature to summarise which training formats or methods had been described in FCV settings, and to describe how often such training was associated with success in these settings. Key effectiveness outcomes were: knowledge, skills, compliance, case counts or case mortality while training delivery was summarised according to key features such as format, duration and delivery mode.

## Methods

PROSPERO registration number is CRD42023472400. We originally planned to undertake a systematic review but later realised that answering our research question was better suited to a scoping review format, where evidence is summarised narratively with respect to creating a comprehensive overview of evidence rather than obtaining evidence to be evaluated for effectiveness. There were two other notable deviations from protocol: we did not use the Covidence platform and we decided to develop and apply a customised quality assessment (QA) checklist instead of originally listed QA instruments. This article is one of several outputs arising from the same protocol.

### Population

Training programmes had to take place in FCV settings or for staff about to be deployed to FCV settings. Fragile or vulnerable settings were defined as being in countries that were designated as low income or lower-middle income by the (World Bank 2022 classification; [8]). Conflicted-affected settings were determined using reader judgement for individual studies, and had to feature concurrent with the training and care delivery, high threat of armed violence or civil unrest. Participants had to be health care professionals (HCPs), social care staff, student or trainee HCPs or trainee social care staff working in an FCV setting. If in doubt about whether the participants qualified, we deferred to World Health Organisation occupational definitions [9]. Voluntary carers such as family members or community hygiene champions as targets were excluded. Eligible interventions could be described as training or education related to any aspect of IPC outcomes.

### Intervention

The training programme could be any training or education that was delivered in a response phase (when there was a concurrently present infectious disease threat) or in the readiness phase [5], when there was high risk that the infectious threat would become present in the clinical environment within six months, such as soon after Covid-19 was declared to be a public health threat of international concern in January 2020.

### Comparator

Comparators were either the same cohort measured at baseline or a contemporaneous cohort in same setting who did not receive IPC training.

### Effectiveness outcomes

Changes in individual knowledge, skills, adherence (compliance or practice), case counts or mortality related to infection were primary effectiveness outcomes. These were chosen because preliminary searches suggested they were commonly reported outcomes in the likely literature. Most of these were immediate benefits that could result as soon as training was completed. We also included case incidence and infection-related mortality as primary outcomes because we knew from preliminary literature searches that these were often the only specific outcomes reported after IPC training. Secondary outcomes (data only collected from articles with at least one primary outcome) were attitudes, acceptability of the training, self-efficacy, confidence, trust in IPC, awareness, index-of-suspicion, ratings for value or relevance of the training, objectives of the training, lessons learned about training needs or recommendations about training needs to be addressed in similar subsequent training programmes.

Outcomes could be objectively- or self-assessed. We wanted to extract outcomes that could be most comparable between studies (not adjusted for heterogenous covariates) and that were objectively assessed rather than self-reported, if possible. Hence, objectively assessed outcomes were extracted and are reported if both objectively- and self-assessed outcomes were available, else self-reported outcomes were extracted and are reported. We extracted and report unadjusted outcomes where available, but adjusted results after post-processing (such as using regression models) were extracted if no unadjusted results were reported.

### Inventory and description of training methods

Specific aspects of how training was delivered were key to understanding the potential that each training programme might have to achieve replicable results elsewhere. We used an iterative process with an expert working group giving advice to develop a list of training features such as setting, duration, target participants and programme design (see list below). These categorisations are not presented as definitive but rather they were pragmatically determined attributes for what information could be gathered in the eligible studies and that directly inform how replicable each education programme was, and how generalisable its results might be in other settings/with other target participants. We extracted information from the studies to categorise the training that they described according to the below features. Multiple answers were possible for many of these features. “Unclear” or “Mixture” were possible answers, too.

*Where (location)*: Off-site without real patients; in house but not while caring for patients; on the job training (during patient care).

*Length* of the training session(s): such as 1 h on one day, or 6 sessions over 8 weeks, etc.

*When (timing with respect to possible threat)*: Pre-deployment to clinical environment; in post or as continuing professional development.

*Mode (of delivery)*: 3 options which were: face to face; blended (a mix of face to face and online) or hybrid (face to face with opportunity for some participants to join in remotely); only digital: e.g. digital resources uploaded to an USB stick or online via an online platform, either synchronous or asynchronous.

*Broad occupational category receiving the training*: Clinical frontline staff; trainers who were expected to directly train others; programme overseers or senior managers.

*Specific occupations receiving the training*: Nurses, doctors/physicians, others.

*Learning group size*: Individual or group.

*Format*: Workshops; courses; seminars/webinars; mentoring/shadowing; e-learning; e-resources, other.

*Methods*: Didactic instruction/lectures/audio-visual presentations; demonstrations/modelling; discussion/debate; case studies or scenarios; role play or clinical practice simulations; assessment or exams with formative assessment; hands-on practice / experience; games; field trips or site visits; virtual reality or immersive learning; repeated training; shadowing; other.

### Additional inclusion and exclusion criteria

We included scientific studies with concurrent comparison groups (CCT or RCT) where post-training outcomes were reported for both arms and pre-post studies where both baseline and post-training measurements of a primary effectiveness outcome were reported. Clinical cases, case reports, cross-sectional studies, letters to the editor, editorials, commentaries, perspectives, technical notes, and review summaries were excluded unless they reported baseline and post-training eligible effectiveness outcomes. Studies must have been published in 2000 or later. Infectious biological entities could be bacteria, viruses, protozoa or funghi, but not complex multicellular organisms (like mites or lice).

Studies could be published in any language that members of the team could read or translate to coherent English using Google Translate. Training in infection prevention and control had to be applicable to a clinical or social care environment for humans. Non-residential care settings (such as daily childcare facilities) were excluded. Studies about controlling infection risks from or to animals or risk reduction in non-clinical environments (such as removing mosquito breeding sites) were excluded.

We wanted to focus on IPC training that related to individual action and could result in immediate benefits and in clinical not community environments. For this reason, we excluded interventions or outcomes that related to: forms of patient care (e.g., anti-viral treatment) that might hasten end of infectious period; vaccination programmes; surveillance; availability of personal protective equipment (PPE) or other resources that reflect institutional will and opportunity as much as any individual action; testing strategies or protocols or actions to speed up test results or screening patients for infection. Also excluded were training programmes in environmental management outside of the clinical/care environment with exception for waste management generated within clinic and managed on site which might include some outdoor/away from clinic/care location handling and disposal decisions.

Eligible studies had to report at least one of our primary outcomes so that we could summarise the evidence base about which training methods linked to evidence of effectiveness. To focus on the response and readiness phase of emergencies, we excluded studies where the primary outcome was only measured > 12 months after training started (i.e., quality improvement reports).

### Searches

MEDLINE, Scopus, LILACS were searched on 9 October 2023 with the search phrase (Scopus syntax):

(“infection-control”[Title/Abstract] or “transmission”[Title/Abstract] or.

“prevent-infectio*”[Title/Abstract]).

And.

(“emergency”[Title/Abstract] or “epidemic”[Title/Abstract] or “outbreak”[Title/Abstract]).

and.

(“training”[Title/Abstract] or “educat*”[Title/Abstract] or “teach*”[Title/Abstract]).

Included studies in a recent and highly relevant systematic review [2] were also screened. Initially included studies from those search strategy steps were then subjected to forward and backward citation searches to look for additional primary studies.

### Screening

After deduplication, two authors independently screened all studies found by the search strategy, recording decisions on MS Excel spreadsheets. All studies selected by at least one author had full text review for final decision about inclusion.

### Quality assessment

We assess quality indicatively and with regard to usefulness of the studies to inform development of future IPC training programmes in relevant settings. The focus was on two broad domains that informed A) how replicable the training programme was, as described; B) how biased its results were likely to be. Our protocol planned to apply the Cochrane Risk of Bias 1.0 for trials (ROB.1) and Newcastle Ottawa Scale (NOS) tools to undertake quality assessment for pre-post study designs. However, we realised that neither of these tools captured whether the original research had reported sufficient details to make the original training programme replicable. Another problem is that the judgements arising from the RoB.1 and NOS would not be strictly comparable, given the different assessment criteria. Other existing quality checklists that we are aware of that were suitable for each of trials, cohorts or pre-post study designs had the shortcomings of only capturing replicability or bias in apparent effectiveness (not both), and tending to be suitable for only one study design. Some checklists (eg The Cochrane Risk of Bias 2.0 tool [10] or Mixed Methods Appraisal Tool [11]) require more resources to operationalise than we had or that was required for a scoping review. Instead, we devised and applied an indicative quality checklist that comprised 14 simple questions with possible answers that were “yes, no or uncertain” using specific predefined criteria for deciding each answer. Our checklist is available as File S1. These questions were modified from suggested questions in the USA National Institutes of Health assessment checklist for pre-post designs [12]. Applying a single quality assessment tool across multiple study designs had the further advantage of facilitating comparability with regard to identifying relative informativeness for future effectiveness evaluation and training programme design. The answers were scored as 1 point per yes answer, so maximum score (for least biased and most replicable studies) would be 14. We interpret the overall quality assessment results as follows: ≥ 11/14 = most informative, 8–10 = somewhat informative, ≤ 7/14 least informative. The quality assessment results are reported quantitatively and narratively. Subdomains for replicability and other bias (generalisability) scores are reported separately.

### Data extraction and interpretation (selection and coding)

These data were extracted: author of the study, year of publication, study country, study design, sample size in comparator arms, relevant infectious diseases (that author identified), primary outcomes, secondary outcomes. With regard to training delivered, we also extracted information about any needs assessment that was undertaken, training objectives and any statements about lessons learned or what should be addressed in future design of such programmes or in research. One author extracted data which was confirmed by a second author. Results are reported quantitatively (counts of studies with any particular training aspect) and narratively for needs assessment, objectives and lessons learned.

To interpret likely usefulness, we prioritise higher scores (for informativeness), but also consider study design, with trials presumed to have less biased results with regard to effectiveness outcomes. We address potential differences that were monitored or observed between knowledge, skills or practices with respect to the training attributes. For instance, were outcomes assessed immediately after training (within 1 day) as opposed to (ideally) observed and assessed independently at least three weeks later, which would suggest knowledge, skills and/or practice retention. We also highlight when training applicable to conflict-affected settings was delivered in that same conflicted-affected setting or prior to entry to the setting (such as for military personnel deployed overseas).

## Results

**Fig. 1:**
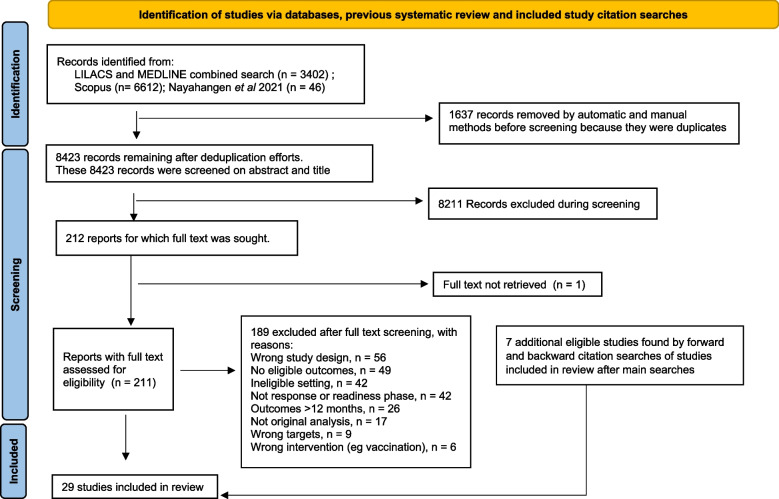
Selection procedure for eligible studies

Figure 1 shows the study selection process. 29 studies were included. Extracted data for each study are in File S2. Almost all (*n* = 27) were pre-post design; 2 were experimental studies [13, 14]. Table 1 lists summary information about the included studies. Seven reports described training delivered in single low-income countries, 19 studies described training in single lower middle income countries. Two articles described IPC training for staff in context of conflict-affected settings, either in the USA prior to military deployment [15] or in the affected setting during a period of civil unrest (in Haiti in 2010; [16]). Two studies [17, 18] described training using a common core curriculum in multiple African countries (mix of low and lower middle income). The most represented countries were India (4 studies) and Nigeria (6 studies). Nine studies were about Ebola disease, 14 related to controlling Covid-19. Other studies addressed cholera (*n* = 2), antimicrobial resistant organisms (*n *= 3) and tuberculosis (*n* = 1). Clinical environments were most commonly described as hospitals (*n* = 9) while twelve studies described programmes for staff working in multiple types of health care facilities. 21 studies were undertaken in response phase, two in readiness phase and six in mixed readiness/response phases. Nurses were the most commonly specified type of health care worker (mentioned in 24 studies). In Table 1, higher scores for knowledge, attitudes, practices or skills were the better clinical outcomes unless otherwise stated. Some additional outcome information for LN Patel, S Kozikott, R Ilboudo, M Kamateeka, M Lamorde, M Subah, F Tsiouris, A Vorndran, CT Lee and C of Practice [18] and N Zafar, Z Jamal and M Mujeeb Khan [19] are in the original studies but could not be concisely repeated in Table 1. Most articles reported statistically significant (at *p* < 0.05) improvements in outcomes after training. A notable exception is OO Odusanya, A Adeniran, OQ Bakare, BA Odugbemi, OA Enikuomehin, OO Jeje and AC Emechebe [20] who attributed a lack of improvement after training to very good baseline knowledge, attitudes and practices.

**Table 1:** Studies included in review

First author, publication year and country	Dates when outcomes were monitored	Outcomes available	Comparator primary outcome results (scores)	Intervention group primary outcome results (scores)
***Covid-19***				
Das 2022 [21] India	Mar 2020 – Jun 2021	Compliance, *Mean %*	Compliance ~ 62%	Compliance ~ 78%
Etebarian 2023 [22] Iran	Sep-Nov 2021	Scores in Knowledge Attitude Practice *Mean (sd)*	Knowledge 7.78 (1.58) Practice 50.75 (5.79) Attitude 43.13 (3.86)	IMMEDIATELY POST Knowledge 10.66 (0.89) Practice 56.81 (3.19) Attitude 45.28, 2.39 2 MONTHS POST Knowledge 11.56 (0.57) Practice 59.40 (0.93) Attitude 45.88 (1.31)
Jafree 2022 [14] Pakistan *trial	Before Feb 2022	Knowledge, IPC practices (compliance) *Mean (sd)*	IPC PRE 31.47 (1.0) POST 33.25 (3.14) Knowledge PRE 31.44 (1.29) POST 33.13 (3.47)	IPC PRE 31.77 (0.55) POST 34.36 (2.68) Knowledge PRE 31.60 (0.79) POST 34.97 (6.13)
Odusanya 2022 [20] Nigeria	Before June 2022	Scores in Knowledge, practice, attitudes *Mean (sd)*	Knowledge: 28.48 (2.24) Attitude 34.44 (3.72) Practice 33.95 (3.13)	Knowledge 28.52 (1.92) Attitude 34.83 (3.84) Practice 34.60 (2.73)
Otu 2021 [23] *Training Nigeria*	30 Mar - 20 Jun 2020	Knowledge *Mean (sd)* Satisfaction with course agreement	53.92 (11.72)	73.55 (12.17) “I learned skills that I can use at work”: 97% agreed or strongly agreed
Otu 2021 [24] *Introduction Nigeria*	Before Mar 2021	Knowledge *Mean (sd)*	47.5 (9.4)	73.1 (10)
Patel 2021 [18] 22 African countries	Unclear, likely 2020- 2021	%HCW cases in 8 countries % of observations (21–1281 each country) that surfaces were disinfected at least twice a day *Mean (sd)*	%HCW cases in 8 countries May 2020: 1.2%, June 2020: 23.7% Angola 70 (8.4) Eswatini 70 (8.4) Lesotho 100 (0) Liberia 31.1 (5.4) Malawi 71.4 (7.0) Nigeria 61.8 (1.4) Uganda 55.1 (2.2) All 8 countries 59.8 (1.1)	%HCW cases in 8 countries November 2020: 1.9% Angola 93.3 (4.6) Eswatini 100 (0) Lesotho 100 (0) Liberia 95.9 (2.3) Malawi 92.9 (3.9) Nigeria 68.1 (1.3) Uganda 86.0 (1.5) All 8 countries 75.4 (0.9)
Perera 2022 [25] Sri Lanka	Mar-Apr 2021	Knowledge scores *Mean*	Physicians 82.76 Nurses 83.29 Midwives 75.39 Support staff 71.20	Physicians 88.30 Nurses 89.71 Midwives 84.14 Support staff 77.15
Rao 2021 [26] India	Mar-Jun 2020	Skills *in specific domains* *Mean (sd)*	*Covid19 prevention skills* Nurses 10.0 (2.7) Paramedics and Non-medical staff 9.8 (0.8) *Environmental sanitation, handling cadavers* 6.6 (2.6) *Ambulance disinfection* 7.7 (2.9) *Precautionary measures around food* 7.1 (2.4)	*Covid19 prevention skills* Nurses 12.1 (3.1) Paramedics and Non-medical staff 11.4 (2.4) *Environmental sanitation and handling cadavers* 12.8 (1.8) *Ambulance disinfection* 13.6 (1.9) *Precautionary measures around food* 13.4 (1.8)
Shehu 2023 [27] India	April 2020	COVID-19 IPC Capacity (custom assessment list) % correct scores by geographic zones *Mean % correct (sd)* Satisfaction with course ratings (Likert scale, 0–10) *Mean (sd)*	*IPC Score by Zone (Z)* Z1 54 (5) Z2 64 (6) Z3 65 (6) Z4 63 (6) Z5 58 (6) Z6 63 (7)	*IPC Score by Zone (Z)* Z1 64 (8) Z2 70 (8) Z3 66 (7) Z4 70 (7) Z5 65 (7) Z6 70 (7) *Format was appropriate* 7.2 (0.83) *Content was valuable* 10.0 (0)
Shrestha 2020 [28] Nepal	Mar 30–Jul 30 2020	Self-reported skill or confidence, *Median (IQR)*	Triage confidence 1 (1–2) Triage skill 1 (1–2)	Triage confidence 4 (3–4) Triage skill 4 (3–4)
Thomas 2022 [29] Nigeria	6 Apr-1 Jun 2021	Knowledge gains *Mean answers correct* Satisfaction with training (Likert score/10)	Knowledge 29%	Knowledge 54% Satisfaction 8.95/10
Tsiouris 2022 [17] 11 African countries	Jun-Oct 2020	Combined knowledge and skills score *Mean (sd)*	Angola 0.57 (0.1) Burundi 0.27 (0.11) Kenya 0.72 (0.14) Lesotho 0.59 (0.14) Malawi 0.56 (0.11) Rwanda 0.59 (0.11) S. Leone 0.54 (0.14) Zambia 0.62 (0.12) Total 0.59 (0.15)	Angola 0.68 (0.10) Burundi 0.69 (0.10) Kenya 0.89 (0.07) Lesotho 0.69 (0.12) Malawi 0.74 (0.11) Rwanda 0.73 (0.10) S. Leone 0.70 (0.13) Zambia 0.70 (0.10) Total 0.73 (0.13)
Zafar 2020 [19] Pakistan	2020 before July	21 individual domains, encompassing: Knowledge Awareness Adherence Confidence *% answers correct*	Knowledge about precautions 87.5 Know how to donn PPE 50.3 Know how to doff PPE 47.8 Usage of sanitizer or hand washing 97.5 Observance of source control measures 31.8 Awareness 24.3 Consider self to be prepared 38.8	Knowledge about precautions 97.5 Know how to donn PPE 92 Know how to doff PPE 65 Usage of sanitizer or hand washing 100 Observance of source control measures 43.5 Awareness 58.8 Consider self to be prepared 81.5
***Ebola disease***				
Bemah 2019 [30] Liberia	Sep 2014 – Mar 2015	HCW case incidence *%* Knowledge *Median (IQR)*	HCW infection rate: 9% Knowledge: 54 (35–75)	HCW infection rate 1% Knowledge 82 (70–99)
Bazeyo 2015 [31] Uganda	Likely 2014	Knowledge, *% with pass scores, by locality*	Arua 57 Busia 59.5 Kasese 68 Kisoro 66.5 Kabale 77.5 Tororo 71.5	Arua 70 Busia 66 Kasese 76 Kisoro 75 Kabale 87 Tororo 88.5
Carlos 2015 [32] Philippines	28 Oct-13 Nov 2014	Knowledge, Confidence Acceptability of training *Mean (IQR)*	Knowledge 7 (6–8)	Knowledge 9 (8–9)
Jones-Konneh 2017 [33] Sierra Leone	Sep 2014 – May 2015	#Cases	About 580	About 45
Kabego 2023 [34] DRC	Dec 2018 to Feb 2019	Practices *IPC mean scores, by category of facility*	Category 1, 55.8 Category 2, 52.9 Category 3, 33.8 Category 4, 17.6	Category 1, 82.3 Category 2, 85.2 Category 3, 76.4 Category 4, 79.4
Oji 2018 [35] Liberia	Apr-Oct 2015	Compliance, including with 8 component checklist (MST) *% correct*	MST 75% Allocated Isolation space 6% Adequate waste/sharps management 60%	MST 90% Allocated Isolation space 30% Adequate waste/sharps management 70%
Otu 2016 [36] Nigeria	Oct 2014 pre Feb 2015 post	Knowledge, practices, attitudes *Mean score or mean frequency practice observed %*	Total knowledge 0.616 EAT score 61.2 Handwashing 0.95 Washing surfaces 0.94	Total knowledge 0.682 EAT score 68.2 Handwashing 0.97 Washing surfaces 0.965
Ousman 2019 [37] DRC	Jun-Jul 2018	IPC scores, based mostly on practices and facility type *Mean (sd)*	Hospitals 8 (2.82) Medical centres 4 (1.29) Health centres 4 (1.20) All HCFs 4.41 (1.88)	Hospitals 50 (11.92) Medical centres 39 (15.57) Health centres 36 (14.28) All HCFs 39.51 (14.87)
Soeters 2018 [38] Guinea	Oct-Dec 2014	Knowledge and skills combined score *Median (IQR)*	Frontline HCW 17 (12–21) IPC supervisor 23 (20–27) IPC trainers 23 (20–24)	Frontline HCW 25 (22–28) IPC supervisor 28 (27–29) IPC trainers 28 (27–29)
***Anti-microbial resistant organisms***				
Crouch 2010 [15] USA pre deployment to conflict zone	2008–2009	Knowledge score *Mean (range)*	65% (range 38–88%)	86% (range 71–100%)
El Sokkary 2020 [39] Egypt	Jun 2016—Oct 2017	#Cases	61	10
Wassef 2020 [40] Egypt	Aug 2018 -Feb 2019	#Cases	Incidence was 49/177, or 0.2768	Incidence was 14/93, or 0.1505
***Cholera***				
Ahmed 2011 [41] Zimbabwe	2008–2009	#Cases, Mortality	7378 cases 332 deaths implied CFR 4.5%	22 cases 0 deaths implied CFR 0%
Tauxe 2011 [16] Haiti	Nov 2010—Apr 2011	Case fatality rate	4%	< 1%
***Tuberculosis***				
Sharma 2021 [13] India *trial	Aug-Oct 2019	Knowledge or Attitude scores *Mean (sd)*	BASELINE Knowledge 10.7 (3.1) Attitude 9.8 (1.8) POST Knowledge 12.4 (4.4) Attitude 9.9 (1.8)	BASELINE Knowledge 11.7 (3.2) Attitude 9.3 (1.8) POST Knowledge 18.2 (5.4) Attitude 10.3 (1.8)

EAT = Ebola attitude test (scored), HCW = Health care worker, IPC = infection prevention and control, sd = standard deviation. 14 additional relevant outcomes were reported in Patel et al. 2021 [18] and Zafar et al. 2020 [19]. (*trial) Study was a trial not pre-post design

Outcomes were assessed immediately after training ended in 14 studies; assessment point was unclear in two studies. Other outcome assessments (*n* = 13 studies) took place between 1 week and 6 months after training finished (especially with respect to case counts or mortality). Because almost all studies reported outcome benefits, studies with delayed assessment cannot be said to have achieved greater benefits.

Needs assessment was described in most studies (*n* = 27). For instance, C Carlos, R Capistrano, CF Tobora, MR delos Reyes, S Lupisan, A Corpuz, C Aumentado, LL Suy, J Hall and J Donald [32] stated that “Although briefings for health care workers (HCWs) in Ebola treatment centres have been published, we were unable to locate a course designed to prepare clinicians for imported Ebola virus disease in developing country settings.” HM Soeters, L Koivogui, L de Beer, CY Johnson, D Diaby, A Ouedraogo, F Touré, FO Bangoura, MA Chang and N Chea [38] cited widespread evidence that there was a high transmission rate to health care workers within Ebola Treatment centres to justify the need for IPC training in these settings. S Ahmed, PK Bardhan, A Iqbal, RN Mazumder, AI Khan, MS Islam, AK Siddique and A Cravioto [41], A Das, R Garg, ES Kumar, D Singh, B Ojha, HL Kharchandy, BK Pathak, P Srikrishnan, R Singh and I Joshua [21] and MO Oji, M Haile, A Baller, N Trembley, N Mahmoud, A Gasasira, V Ladele, C Cooper, FN Kateh and T Nyenswah [35] describe that expert observers identified deficiencies in existing IPC practices and developed training based on those observations. Independent observations of training needs were formalised as a cross-sectional survey of dental student IPC knowledge in A Etebarian, S Khoramian Tusi, Z Momeni and K Hejazi [22], and by applying a validated IPC checklist in L Kabego, M Kourouma, K Ousman, A Baller, J-P Milambo, J Kombe, B Houndjo, FE Boni, C Musafiri and S Molembo [34].

All studies stated specific training objectives and gave at least some information about the specific topics and curriculum. Objectives statements mentioned improvement (*n* = 10 studies), knowledge (*n* = 7), safety (*n* = 6), attitudes (*n* = 3), increasing capacity or skills (*n* = 6), and development (*n* = 1). Examples of other objectives statements were to “teach the basics” [41] or “to cover the practical essentials” [16]. Training content and delivery were often highly adapted for local delivery [23–25, 28, 29, 32, 33, 36, 38, 41]. Training materials were entirely or mostly derived from published guidance in some studies [16, 19, 34, 35, 37]. F Tsiouris, K Hartsough, M Poimboeuf, C Raether, M Farahani, T Ferreira, C Kamanzi, J Maria, M Nshimirimana and J Mwanza [17] and LN Patel, S Kozikott, R Ilboudo, M Kamateeka, M Lamorde, M Subah, F Tsiouris, A Vorndran, CT Lee and C of Practice [18] both report that training delivery methods were highly adapted and variable, but developed using the same core course content about Covid-19 in 11 or 22 African countries. Other studies were unclear about how much of their programme was original and how much relied on previously published guidance and recommendations [13, 15, 20, 21, 24, 26, 27, 30, 31, 39, 40].

Counts of training locations were: ten off-site; seven on-site but not during patient care; nine were a mix of learning locations; three had unclear locations relative to clinical facility location. Among the 21 studies that described the specific cumulative duration of training sessions, median training duration was 24 h (typically delivered over 3 consecutive days), ranging from about 15 min to 8 full days. Most studies (*n* = 21) described training where it was clear that many or most participants were in post, 3 studies clearly described training being provided prior to deployment, another 5 training programmes had mixed or unclear timing with regard to deployment. Twelve studies described training that was delivered only in person, 9 studies described purely digital delivery, 7 were blended delivery and 1 programme was unclear whether the training was delivered digitally or in person. In terms of IPC roles, all studies included at least some frontline workers. In addition, six studies were explicitly designed to train people who would educate others about IPC, seven studies reported including facility managers or supervisors among the trainees. 23 studies mentioned nurses specifically among the trainees, 17 studies specifically mentioned doctors or physicians. Other professionals mentioned were cleaners, porters, paramedics, midwives, anaesthesiologists, hygienists, housekeeping staff, lab technicians, medical technologists and pharmacists. Almost half (*n* = 14) of studies were group education; purely individual learning was specified in just one study and others (*n* = 14) were unclear or could be either individual or group learning.

Often training formats or teaching methods were described unclearly. With regard to formats that were described clearly, counts were workshop (*n* = 10), course (22), seminar or webinar (1), mentoring or shadowing (4), e-learning (13) and inclusion of e-resources (14). Counts of studies using specific teaching methods that were described clearly were didactic (23), demonstrations (17), discussion or debate (8), case-studies or scenarios (6), role play or simulations (9), formative assessment (3), hands-on practice (12), site visits (2), repeat or refresher training (5), shadowing (3). Additional teaching methods described specifically were poster reminders, monitoring (active and passive as well as observation), re-enforcement (updating procedure documents, re-assessing, more training), brainstorming, small group work and other visual aids. Many articles described multiple formats or teaching methods that were used as part of the same training programme, hence these categorisations sum up to more than the total count of included studies.

Most studies (*n* = 25) provided some commentary that could be interpreted as “lessons learned” about training methods and delivery. That success of such programmes depends as much on improving mindset or attitude about IPC as teaching other skills or habits was mentioned by at least 6 studies [13, 14, 20, 22, 32, 39]. The merits of capacity building were explicitly reiterated in concluding commentary in seven studies [21, 26, 29–31, 35]. Other aspects repeatedly endorsed (at least three times) in concluding comments in the included studies were the value of IPC champions or leaders [21, 34, 35] the value of training relevant to specific job role [14, 18, 22, 31]; advantages of digital not in-person learning [13, 14, 19, 20, 23]; value of refresher sessions [13, 14, 17, 21, 30, 35] and merits of evaluation beyond the immediate end of the training programme to make sure that benefits were sustained [21, 29, 38, 39]. Regarding lessons learned, Thomas et al. 2022 [29] and Otu et al. 2021 [24] (both Nigerian studies) gave specific details about challenges and benefits of mobile phone digital training delivery, for instance reliance on assumed e-literacy, uncertainty about consistent access to Internet or access to devices with suitable versions of the Android operating system. Four studies [14, 25, 29, 38] listed benefits when training was delivered in participant’s native language(s).

Quality assessment scores are shown in Table 2. Recall that the customised quality assessment evaluation addressed two broad domains: replicability and other biases (other potential for generalisability), with results interpreted as usefulness of the study to inform future design of similar IPC training programmes. The quality assessment found that replicability potential was not high overall, with an average score of 3.7/6. There was insufficient easily available information (score was < 4 of 6 replicability domains in QA checklist) to undertake the same intervention again for 11 studies, while replicability was relatively high (≥ 5/6) for 9 studies. The generalisability domain in the quality assessment checklist addressed other factors that may have biased the apparent effectiveness outcomes of each training programme*.* 22 studies scored < 5/8 for generalisability (suggesting they were likely to be at high risk of bias with regard to outcomes reported). Only one study was assessed to be of overall relatively higher quality (quality checklist score ≥ 11/14) and can be considered especially (“most”) useful for informing design of such IPC training in future. Shreshtha et al. [28] had a pre-post design and is especially thorough in describing training in intubation and triage protocols in Nepal to prevent Covid-19 transmission. The two controlled trials included in our review [13, 14] both scored below 11 (10/14) in the quality assessment because they had unclear information about how many participants were assessed and did not provide specific training or assessment materials. There was minimal or no difference in most outcome improvements between arms in one of the trials (Jafree et al. 2022; [14]), but statistically significant greater improvement in outcomes, especially knowledge, in the active intervention arm, in the other trial. (Sharma et al. 2021; [13]). This number of experimental trials was small (*n* = 2) and they described fairly different format training programmes for different diseases.

**Table 2.**
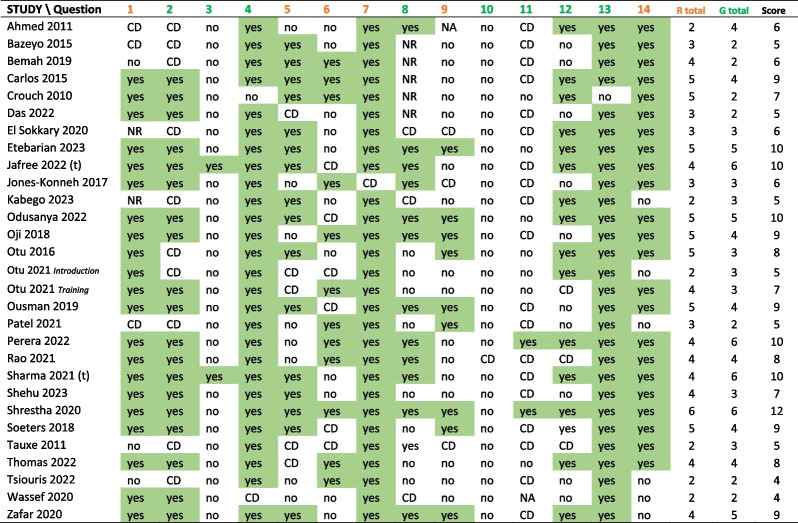
Quality assessment for included studies and likelihood of being useful to inform future IPC training [41, 31, 30, 32, 15, 21, 39, 22, 14, 33, 34, 20, 35, 36, 24, 23, 37, 18, 25, 26, 13, 27, 28, 38, 16, 29, 17, 40, 19]

See File S1 for questions and answer criteria. (t) = trial; all other studies were pre-post design. Orange number = replicability question, Green number = generalisibility question

## Discussion

The evidence available is difficult to interpret because of incomplete reporting and lack of specific descriptions. Training delivery was often vaguely described, or even explicitly described as highly diverse while relatively few pathogens were addressed. Only two moderate size (*n* = about 200 in each) experimental trials were found which is insufficient for making broad conclusions about effectiveness. It seems likely that many possible training methods can successfully improve HCW knowledge, skills, attitude, practices, etc. We note that there is unlikely to be definitive evidence in favour of or against specific training methods due to lack of thorough description of training methods in addition to lack of robust study designs (very few clinical trials). Lack of specificity about which aspects of training were least or most beneficial may hinder successful development of future training programmes. Lack of controlled trials and generally poor description of any training programmes that existed prior to implementation of the programmes described in pre-post studies means that we can’t discern if training was effective because of how it was delivered or because relevant training had never been given previously. It seems clear that there is huge opportunity for design of well-run controlled trials in IPC training delivery. A controlled trial could be designed and tested with a pre-specified curriculum for a common and recurring type of pathogen (e.g., influenza-like illness or for a specific common anti-microbial resistant organism), but with 2 or more delivery formats pre-approved with institutional review bodies, and thus ready to be implemented when a relevant crisis arose. Suitable outcomes to include in the trial design would measure aspects of knowledge, practices, skills, confidence and awareness. Complexity-informed evaluation strategies [42] are likely to be desirable in fragile, conflict-affected or vulnerable settings, too. (Nayahangan et al. 2021; [2]) recommended that medical training be more standardised during viral epidemics. We did not find evidence to show that universally formatted IPC training programmes are optimal in FCV settings. We have, however, provided information that can be used to begin to assess effectiveness of training programmes that are either universally formatted or more highly locally adapted.

Only two of our studies described training that was applied in conflict-affected settings; one of these [15] described training that was also delivered prior to worker arrival in the conflict-affected setting. We judge that these two studies are too few and too heterogenous to pool, so we cannot draw broad conclusions about training delivery and benefits in a conflict-affected area context or in a high resource setting prior to deployment.

Other researchers have systematically described many key issues that affect effectiveness of IPC training in low resource or conflict-affected settings. For instance, Qureshi et al. 2022 [43] undertook a scoping review of national guidelines for occupational IPC training. They audited how up to date such guidelines were. They identified key deficiencies, especially in LMIC countries with regard to the most recent best recommended practices in evaluation and adult learning principles. A global situational analysis undertaken in 2017–2018 [1] concluded that although nearly all countries audited had relevant national training guidelines in IPC, there was far less training of HCWs taking place, less surveillance and lower staffing levels in lower-middle and lower-income countries (World Bank classifications) than in upper-middle and high income countries.

Data and analyses have been undertaken to specifically describe challenges and potential strategies to meet those challenges, when undertaking IPC in conflict affected settings [44] or low and middle income countries dealing with a specific disease [e.g., tuberculosis; 45]. These studies are fundamentally qualitative in design and narrative, so while they provide insight, they do not lead to confident conclusions about which if any training methods are most likely to be successful. There is a dearth of experimental evidence in lower-middle and lower income countries. The Covid-19 pandemic especially focused interest on IPC guidelines for respiratory infection prevention. A review by Silva et al. 2021 [46] of randomised controlled trials that tried to improve adherence to IPC guidelines on preventing respiratory infections in healthcare workplaces included 14 interventions, only one of which was not in a high income setting [in Iran; 47], and all were in arguably undertaken in preparation phase (not response or readiness).

### Limitations

Although we included incidence and mortality as primary outcomes, these outcomes are often not immediate benefits from good IPC training and thus are problematic indicators of IPC success. Case incidence is highly dependent on local community prevalence of relevant pathogen(s), while mortality rates often reflect quality of medical care available in addition to population awareness and subsequent timing of presentation. Our search strategy was not tested using eligible exemplar studies, nor did it include controlled vocabulary which might have found additional eligible studies. We did not rigorously determine risk of bias in each of the few trials available. We did not explicitly look for evidence of publication bias [48] in this evidence group, but we suspect that the near total absence of any information about failed interventions biases what we can say with confidence about truly successful training formats and methods.

A key limitation when we graded the studies for likely usefulness is that we did not attempt to contact primary study authors to obtain more information or specific training materials. Additional materials are likely to be available from most of the primary study authors and would boost their study replicability and apparent biases. However, such contact could also be a very demanding and not necessarily productive exercise. A broader review than ours could have collected all evidence about any training modalities when delivered in eligible contexts (readiness or response phase in FCV settings), regardless of whether effectiveness outcomes were reported. A review with similar such objectives was published in 2019 [7], which inventoried implementation strategies for IPC promotion in nurses in Sub-Saharan Africa.

We decline to adopt a broad inventorying approach because the information obtained would still lack evidence of effectiveness. We found some studies [e.g., 49] which provided a thorough description of training delivery, but without evaluation of our outcomes and therefore ineligible for inclusion in our review. A broader review than ours would have included grey literature and qualitative studies. Qualitative studies especially provide information about effective communication and leadership, acceptability of training delivery methods, incentives, accountability strategies, satisfaction ratings and barriers to learning [50]. While those are highly relevant outcomes to effective training in IPC, they were removed from the core outcome that is likely to matter most in achieving good IPC, which is consistency of desired practices.

## Conclusion

Our conclusions are limited because of the mediocre quality of evidence available. Although existing evidence in favour of or against any specific training approach is far from definitive, there is much opportunity to design future studies which explicitly and robustly test specific training formats and strategies.

### Supplementary Information

Supplementary Material 1.

Supplementary Material 2.

## Data Availability

The datasets used and/or analysed during the current study are available from the corresponding author upon reasonable request.
