# Supplementary material for: Emergency infection prevention and control training in fragile, conflict-affected or vulnerable settings: a scoping review
Source: BMC Health Serv Res. 2024 Aug 16;24:937. doi: 10.1186/s12913-024-11408-y (PMC11328436; doi:10.1186/s12913-024-11408-y)
Supplement: Supplementary file 1 — Supplementary Material 1. [file 12913_2024_11408_MOESM1_ESM.docx]

Questions especially oriented toward if there is replicability in intervention deliverability

Questions oriented towards additional confounders and other biases (which will contribute to lack of generalisability or distorted observed effect between intervention as delivered and measured outcomes)

| **Criteria** |  |
| --- | --- |
| 1. Were eligibility/selection criteria for the study population clearly described?  YES = if new investigators were to conduct this study again, they would know whom to recruit, including from what setting. |  |
| 2. Were the participants in the study likely to be representative of those who would be eligible for the test/service/intervention in the general or clinical population of interest?  Researcher judgement, YES = The participants were not obviously likely to be unrepresentative of the population in which the intervention was applied. |  |
| 3 Was the study design a controlled trial (contemporaneous comparator groups)? |  |
| 4. Was the sample size sufficiently large (at least 15 in each arm (trials) or combined total (BAC) at least 30) to provide confidence in the findings? (Yes at least 15 or combined at least 30, else No answer) |  |
| 5. Was the test/service/intervention delivered (as reported) consistently across the study population?  Yes = constant application of same programme (Y) vs. agile development (N).  6. Are any specific materials available to make the training replicable?  YES=eg., The article provides a list of items on curriculum, or link where to find or at least one slide, video, etc. |  |
| 7, 8, 9 Were the outcome measures clearly described, validated, or available to replicate?  **7. Were outcome measures** clearly described?  YES = described in a broad sense, both concept(s) & strategy for measuring them.  **8. Were Outcome measures validated?**  YES=in some way validated (one of at least one of these options: a Cronbach alpha, or piloting by at least 5 target individuals with revisions considered, or someone else’s previously used tool) - so validated, consistent, reliable.  **9. Is the assessment tool specific?**  **YES =** provided for readers to see and use (eg checklist, test, etc.)? |  |
| 10. Were the people assessing the outcomes blinded to the participants' exposures/interventions? YES = only if they clearly state there was blinding. |  |
| 11. Of those who completed pre-assessment evaluation, did at least 80% of them get reported at post/in full evaluation for most outcomes?  YES = at least 80% of participants got evaluated. |  |
| 12 Were any of the reported concurrent complimentary measures that happened likely to have also changed the primary outcomes?  (analyst judgement, same answer options as most others.)  13 Was the training provided in the FCV setting?  YES if true, and not provided to people elsewhere then deployed to the FCV setting (eg., EVD training in USA but then deployed outside USA).  14 Were the majority (n ≥ 10) of the 18 (total) format and method training choices in preset taxonomy clearly present or not, thus a potentially replicable programme? YES/NO answer |  |

Answer options are Yes, No, CD, cannot determine; NA, not applicable; NR, not reported
